# The organization of biological sequences into constrained and unconstrained parts determines fundamental properties of genotype–phenotype maps

**DOI:** 10.1098/rsif.2015.0724

**Published:** 2015-12-06

**Authors:** S. F. Greenbury, S. E. Ahnert

**Affiliations:** Theory of Condensed Matter, Cavendish Laboratory, University of Cambridge, Cambridge CB3 0HE, UK

**Keywords:** evolution, genotype–phenotype map, robustness, evolvability, RNA

## Abstract

Biological information is stored in DNA, RNA and protein sequences, which can be understood as genotypes that are translated into phenotypes. The properties of genotype–phenotype (GP) maps have been studied in great detail for RNA secondary structure. These include a highly biased distribution of genotypes per phenotype, negative correlation of genotypic robustness and evolvability, positive correlation of phenotypic robustness and evolvability, shape-space covering, and a roughly logarithmic scaling of phenotypic robustness with phenotypic frequency. More recently similar properties have been discovered in other GP maps, suggesting that they may be fundamental to biological GP maps, in general, rather than specific to the RNA secondary structure map. Here we propose that the above properties arise from the fundamental organization of biological information into ‘constrained' and ‘unconstrained' sequences, in the broadest possible sense. As ‘constrained' we describe sequences that affect the phenotype more immediately, and are therefore more sensitive to mutations, such as, e.g. protein-coding DNA or the stems in RNA secondary structure. ‘Unconstrained' sequences, on the other hand, can mutate more freely without affecting the phenotype, such as, e.g. intronic or intergenic DNA or the loops in RNA secondary structure. To test our hypothesis we consider a highly simplified GP map that has genotypes with ‘coding' and ‘non-coding' parts. We term this the Fibonacci GP map, as it is equivalent to the Fibonacci code in information theory. Despite its simplicity the Fibonacci GP map exhibits all the above properties of much more complex and biologically realistic GP maps. These properties are therefore likely to be fundamental to many biological GP maps.

## 1. Introduction

Biological evolution is characterized by the inheritance, mutation and translation of biological information. This information is stored sequentially, in DNA, RNA and protein sequences. Such sequences are more generally referred to as genotypes. Much of biological research investigates in some form or other how specific genotypes translate into biological phenotypes. In recent years, the larger-scale study of genotype–phenotype (GP) mappings has attracted increasing attention, particularly in the context of RNA secondary structure [1–7], which provides a biologically relevant, yet tractable system for the study of entire GP maps. The RNA secondary structure map has yielded a number of insights. Firstly, genotypes vastly outnumber phenotypes: 1.7 × 10^7^ possible sequences of RNA of length *L* = 12 map to just 57 phenotypes, and 1.1 × 10^12^ sequences of length *L* = 20 map to 11 218 phenotypes [7]. Secondly, the distribution of the number of genotypes per phenotype is highly biased. For instance, 95% of all *L* = 20 genotypes map to 10% of all phenotypes [7]. Thirdly, almost all phenotypes can be accessed via a small number of point mutations of the genotypes of any given phenotype. This observation is typically referred to as shape-space covering [1,6,7] and is evidence for the short paths that connect any two phenotypes in the densely connected and highly regular genotype network of point mutations. The structure of the GP map has consequences for evolution, as has also been demonstrated by the study of robustness and evolvability of RNA secondary structure. Wagner [3] introduced quantitative measures of robustness and evolvability that measure the resilience of a phenotype to mutations of its genotypes, and the potential for a phenotype to change into a different phenotype in order to adapt. These quantities can be defined on both the genotypic and phenotypic level. In RNA secondary structure, genotypic robustness and evolvability are negatively correlated. This represents a trade-off that seems inevitable at the genotypic level—a given genotype can either be surrounded by genotypes of the same phenotype (and therefore be robust) or by genotypes of many other phenotypes (and therefore be evolvable) but not both at the same time. In the same system, however, phenotypic robustness and evolvability are positively correlated, demonstrating how biological organisms can be both robust and evolvable at the same time. The reason for this lies in the ‘shape’ of the phenotypes in genotype space. Phenotypes take the form of one or several connected components in the genotype network. These components are often referred to as neutral networks [2] as their edges describe neutral mutations of the genotype, which leave the phenotype unchanged. Some indication of the topological properties of these networks is given by the observation that robustness of a given phenotype scales logarithmically with the size of its genotype network [5,7]. All the above observations have been made in RNA secondary structure, but it has recently been established that most of these properties can also be found across different GP maps, such as the HP model of protein folding [6,7] (where a biased distribution of genotypes per phenotype has been known for some time to exist [8]) and the Polyomino model of biological self-assembly [7,9,10]. This raises the question whether the observed properties are fundamental characteristics of biological GP maps. In this paper, we argue that this is the case, and that these properties are a result of the way in which biological information is organized into sequences that contain distinct regions that code for a phenotype, and non-coding regions that do not. This distinction is of course not clear-cut. Intergenic and intronic DNA may still code for a phenotype, such as microRNAs, for example, and parts of most protein-coding DNA can be mutated without any discernible phenotypic consequences. But it is indisputable that, largely speaking, exonic DNA is mutationally far more constrained than intronic or intergenic sequences. In RNA secondary structure, a bimodal distribution in the neutral mutation rates of constrained and unconstrained sequences has been demonstrated [5,11,12]. These results show that mutations in the loop regions of secondary structure are much more likely to leave the phenotype unaffected than mutations in the stem regions. We consider here a simple model with a genotype that is divided into regions that code for a phenotype, and ones that do not, and show that this model gives rise to all the properties observed in the RNA secondary structure GP map and other GP maps, as outlined above. This provides a strong argument that the fundamental organization of biological information into a series of constrained and unconstrained sequences has profound effects on the structure of biological GP maps, and thus on the translation of genotypes into phenotypes, and the course of biological evolution.

## 2. The Fibonacci GP map

**Figure 1.**
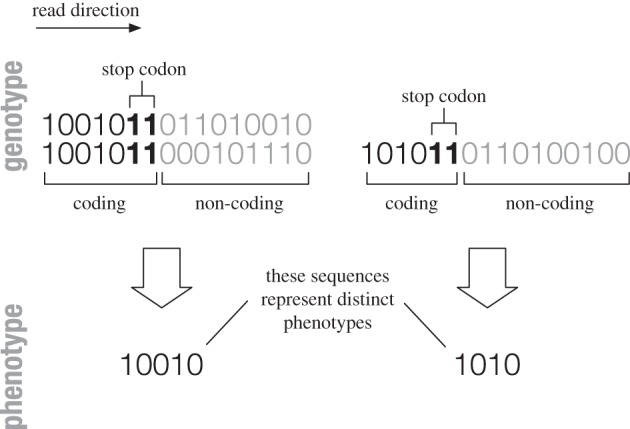
Three examples of genotype sequences, which map to two different phenotypes in the Fibonacci genotype–phenotype map. Reading from the left the sequence is regarded as ‘coding’ up to the first occurrence of the ‘stop codon’ sequence 11. Thereafter the sequence is regarded as ‘non-coding’. Each possible coding sequence represents a different phenotype, whereas the non-coding sequence leaves the phenotype entirely unaffected.

**Figure 2.**
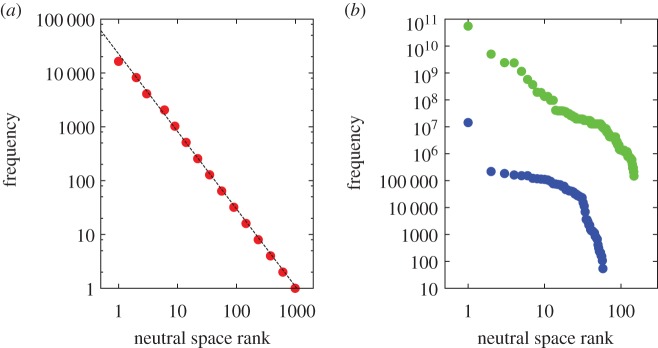
(*a*) The distribution of neutral space sizes in the Fibonacci GP map is highly biased. The rank distribution follows a power law (simulation in red, analytical prediction *kr^*α*^* in black). (*b*) Corresponding distributions for RNA (*L* = 12, blue) and polyominoes (*S*_3,8_, green) follow similarly biased distributions [7].

**Figure 3.**
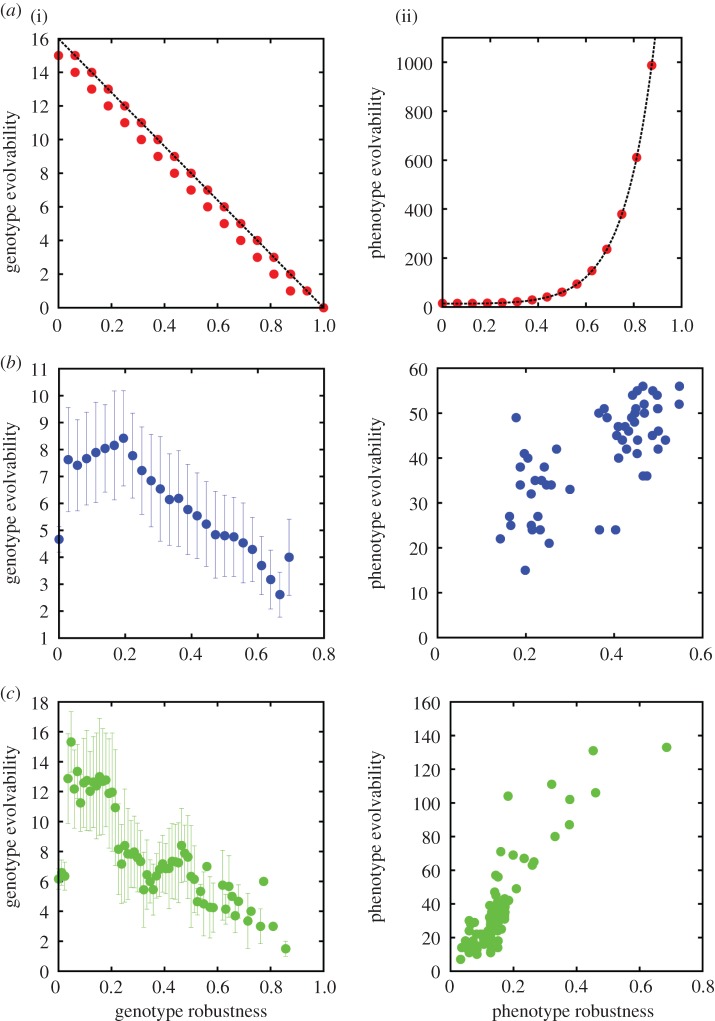
(*a*) Results for the Fibonacci GP map: (i) genotypic robustness is negatively correlated with genotypic evolvability. (ii) Phenotypic robustness is positively correlated with phenotypic evolvability. Analytical results are shown as black lines, computational results in red. The reason for the ‘stepped’ appearance of the left plot is that *e*_g_ can be equal to *l* or *l* − 1, depending on the presence of a second stop codon. (*b*) These relationships mirror the same negative (genotypic) and positive (phenotypic) correlations found in RNA *L* = 12, which are highly statistically significant (*p* < 10^−6^). The data are taken from [7]. (*c*) Polyominoes (here *S*_3,8_) display the same behaviour [7] with high statistical significance (*p* < 10^−6^). For RNA and polyominoes, the genotype evolvability is averaged for a given value of genotype robustness, and the error bars represent the standard deviations of the evolvabilities for a given value of the robustness. The trivial phenotypes, which are unfolded RNA and unbounded or non-deterministic polyomino tile sets have been removed in this figure.

**Figure 4.**
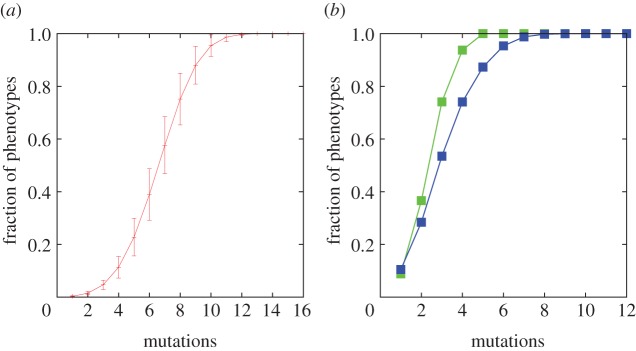
(*a*) The average fraction *f*(*n*) of phenotypes that are *n* mutations away from a given genotype in the Fibonacci GP map. The majority of phenotypes is accessible in a small number of mutational steps. (*b*) Results for RNA (blue) and polyominoes (green) are similar.

**Figure 5.**
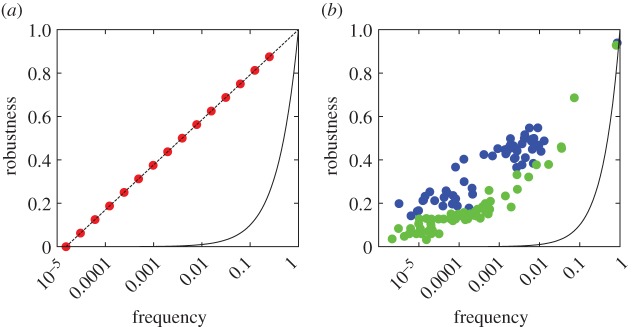
(*a*) The robustness of phenotypes in the model scales logarithmically with the phenotype frequency and lies far above that expected from randomly distributed phenotypes (solid line). (*b*) This represents a similar trend to the robustness of RNA (blue) and polyomino (green) phenotypes as a function of their frequency.

In our model, there is only one coding and one non-coding region, and every distinct sequence in the coding region codes for exactly one phenotype, while the non-coding part of the sequence leaves the phenotype entirely unaffected. Genomes are binary sequences of fixed length in our model, and starting with the first digit the sequence is considered ‘coding’ until a ‘stop codon’ is encountered, after which the sequence is considered ‘non-coding’. Each possible sequence up to the first occurrence of the stop codon uniquely maps to a distinct phenotype. The sequence after the first stop codon, on the other hand, is completely free to mutate, giving rise to the neutral space of the phenotype. For a stop codon sequence of ‘11’, this GP map is equivalent to the Fibonacci code in information theory [13]. This is because the Fibonacci code reads a sequence of binary digits from left to right, up to (and including) the first occurrence of ‘11’ in the sequence. Such a binary sequence **d** forms a Fibonacci code word, and can be mapped to the space of integers by calculating 
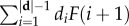
, where *d_i_* is the *i*th digit of **d**, |**d**| is the length of the sequence **d** and *F*(*i*) is the *i*th Fibonacci number, i.e. *F*(l) = 1, *F*(2) = 1, *F*(3) = 2, etc. We therefore term this GP map the ‘Fibonacci GP map’ (figure 1). The 10 shortest coding sequences are given in table 1. Note that sequences without a stop codon do not map to a valid phenotype and are assigned to an ‘undefined’ phenotype, similar to the trivial, non-folding phenotype in RNA, or the UND structure in the Polyomino GP map [7]. Throughout this paper, we will assume a sequence length of *L* = 16 for the genotypes of the Fibonacci GP map. All results are robust, and also hold for sequence lengths other than this. The Fibonacci GP map was implemented in Python through exhaustive enumeration of *L* = 16 genotypes. This enabled us to calculate all the numerical results in this paper, shown as the red data points in figures 2–5. These results are also available as part of the electronic supplementary material. The Polyomino GP map *S*_3,8_ was also enumerated exhaustively, using C++, and is identical to the implementation described in detail in [7]. The RNA secondary structure GP map was exhaustively enumerated using the Vienna package [14] with default parameters, again following the implementation in [7].

**Table 1.** The 10 shortest phenotype sequences in the Fibonacci GP map. The first column gives the phenotype ID in form of an integer. The second column shows the genotype sequence that defines the phenotype. Each genotype sequence corresponds to a Fibonacci code word. The third column shows the Fibonacci number *F*(*n*). The fourth shows the decomposition of the phenotype ID in terms of the genotype sequence, or Fibonacci code word. The last symbol in each sequence (always a one) is ignored. The *i*th symbol represents a contribution of *F*(*i* + 1), so that, e.g. 1011 represents *F*(2) + *F*(4) because the first and third entries of the sequence are ones.

phenotype (*n*)	genotype	*F*(*n*)	decomposition
1	11	1	*F*(2) = 1
2	011	1	*F*(3) = 2
3	0011	2	*F*(4) = 3
4	1011	3	*F*(2) + *F*(4) = 4
5	00011	5	*F*(5) = 5
6	10011	8	*F*(2) + *F*(5) = 6
7	01011	13	*F*(3) + *F*(5) = 7
8	000011	21	*F*(6) = 8
9	100011	34	*F*(2) + *F*(6) = 9
10	010011	55	*F*(3) + *F*(6) = 10

### 2.1. Biased distribution of the number of genotypes per phenotype

The distribution of the number of genotypes per phenotype—or in other words, the size distribution of the neutral spaces—is heavily biased in the case of RNA secondary structure [1,6,7,15] and the Polyomino model [7]. This bias in the GP map can strongly affect evolutionary outcomes [16]. The same biased distribution is also exhibited by the Fibonacci GP map (figure 2), and follows a power law, with few large neutral spaces and many small ones. Analytically, we can confirm this by considering that each coding sequence maps to a distinct integer, representing a distinct phenotype, and that, as this integer increases, the length of the coding sequence increases by one every time a new Fibonacci number is reached. As the distance between two consecutive Fibonacci numbers is simply the preceding Fibonacci number, this means that the number *C*(*l*) of phenotype sequences of length *l* is equal to *F*(*1* − 1). The *n*th Fibonacci number is, to a good approximation given by 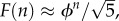 where 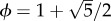 is the Golden Ratio, so that the number *C*(*l*) of different coding sequences of length *l* is
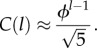
If the total genome length is *L*, the number of genotypes *f*(*l*) that map to a particular phenotype with coding sequence length *l* is 2*^L^*^−*l*^. Hence the number of phenotypes with a neutral space of size *f*(*l*) is *C*(*l*), which means that
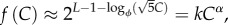
which is a power law, with 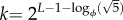 and 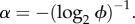 Some studies have plotted the size of the neutral space size against its rank [6,7]. In the Fibonacci GP map, the rank *r* of a neutral space in terms of its size is given by the integer that the coding sequence maps to in the Fibonacci code. As there are *F*(*l*) phenotype sequences of length *l* + 1 and the sum of the first *l* Fibonacci numbers is *F*(*l* + 2) − 1, the number *N*(*l*) of phenotype sequences of length up to and including *l* is given by
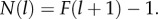


The rank of a sequence of length *l* is the number of phenotype sequences of length up to (but not including) *l*, plus one. In other words, the rank is given by *r* = *F*(*l*), and thus, to a good approximation 
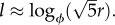
 The size *f* of the neutral space, which is *f* = 2*^L−l^* is therefore related to its rank by
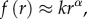
where 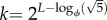 and *α* is the same as above—in other words, another power law, which is broadly what has been found for RNA and polyominoes [1,6,7,15]. These power laws are a direct result of the hypercube-like structure of the point mutation network of genotypes, in which neutral components (connected sets of genotypes of the same phenotype) are hypercube-like subspaces of lower dimensionality. The dimension of these subspaces equals the number of unconstrained bases. The biased distribution of neutral space sizes in RNA exhibits a shallower gradient for larger neutral spaces, and a steeper one for smaller spaces. The reasons for this more complex distribution may lie in the definition of the phenotype, because RNA structures with the same simple loop structure in different positions will constitute different phenotypes. This freedom leads to a larger number of phenotypes with large neutral network sizes than one would get by simply considering the number of constrained and unconstrained sites. It is, however, also a relatively small effect on the order of magnitude of the entire distribution.

### 2.2. Evolvability and robustness

Biological organisms have to be both robust against mutations of the genotype, and also capable of adaptation, and evolvable. On a genotypic level, these two properties are opposed—a genotype can either be robust (surrounded by genotypes of the same phenotype) or evolvable (surrounded by genotypes of many other phenotypes). Wagner [3] showed that while this holds true on a genotypic level, resulting in a negative correlation of genotypic robustness and evolvability, the converse holds true for phenotypes: phenotypic evolvability and robustness are positively correlated. Defined precisely, genotypic robustness is the fraction of neutral mutations per genotype, and genotypic evolvability is the number of distinct phenotypes that are within one mutation of the genotype (and are not the same phenotype as that of the genotype). By contrast, phenotypic robustness is defined as the average fraction of neutral mutations per genotype across a given phenotype. This correlates positively with phenotypic evolvability, defined as the total number of distinct other phenotypes that are within one mutation of any of the genotypes belonging to the given phenotype.

In the Fibonacci GP map, the robustness *r*_g_ of a given genotype g, meaning the fraction of neutral mutations out of all possible ones, is
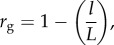
where *l* is the length of the coding sequence. The genotypic evolvability *e*_g_ is simply:
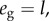
if there is a second stop codon in the sequence, and
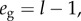
if there is no second stop codon, as any mutation to the first stop codon will lead to the undefined phenotype. The negative correlation between *r*_g_ and *e*_g_ is therefore trivial.

As the genotypic robustness is the same for every genotype of a given phenotype, the phenotypic robustness *r*_p_, being the average of the genotypic robustness *r*_g_ over the phenotype, is equal to *r*_g_:
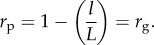


The phenotypic evolvability is the total number of phenotypes that are accessible from the genotypes of a given phenotype via single-point mutations. Recall that we have *F*(*l* + 1) − 1 phenotype sequences of length up to and including *l*. We have two possible mutations of the stop codon. The first mutation turns ‘11’ into ‘01’, which means that the last *L* − *l* + 1 bases in the sequence can be any Fibonacci code sequence of length *L* − *l* + 1, starting with a 1. There are *F*(*l* − 1) such sequences of length *l*, because the sequence has to either start with ‘10’ and then be followed by any Fibonacci sequence of length *l* − 2, or start with ‘11’. The first mutation therefore gives rise to *F*(*L* − *l*) phenotypes. The second possible mutation of the stop codon, turning ‘11’ into ‘10’ simply leads to *F*(*L* − *l* + 1) − 1 phenotypes, because any phenotype sequence of length *L* − *l* can follow. We can also access another *l* − 2 phenotypes by mutating the phenotype sequence before the stop codon. Note that this always leads to distinct phenotypes, regardless of whether a new stop codon is generated by the mutation or not. Counting the undefined phenotype as another phenotype, the total phenotypic evolvability therefore is
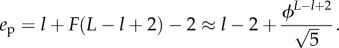


The scaling of phenotypic evolvability *e*_p_ with phenotypic robustness *r*_p_ is therefore
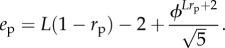


This has a positive gradient if
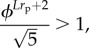
which is always the case, as *L* > 0 and *r*_p_ > 0. Phenotypic evolvability and robustness are therefore positively correlated in the Fibonacci GP map (figure 3), in line with the results observed in RNA secondary structure [3] and the Polyomino GP map [7]. The expression for *e*_p_ offers an important insight into the possible reasons behind the positive correlation of phenotypic evolvability and robustness, as the first term in the equation, *L*(1 − *r*_p_) − 2 would by itself lead to a negative correlation between *e*_p_ and *r*_p_. This term corresponds to mutations of the phenotype sequence. The second term, 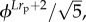 corresponds to mutations of the ‘stop-codon’ sequence, in other words, to mutations of the boundary between coding and non-coding sequence in the genotype. This term provides the positive correlation between phenotypic evolvability and robustness, as the corresponding mutations provide access to much wider range of phenotypes.

### 2.3. Phenotype coverage

Ferrada & Wagner [6] observed that a large fraction of all phenotypes is accessible via a small number of point mutations from almost any genotype. The number of phenotypes accessible from a single genotype via a single-point mutation is the genotypic evolvability *l*. The average length of the phenotype sequence is
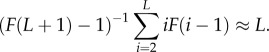


The number of phenotypes accessible through *n* mutations is therefore approximately 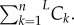 For *n* = *L*/2 this is approximately 2*^L^*^−1^, which is already considerably larger than 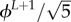 by a factor of 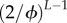 (as 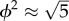). We therefore expect the majority of phenotypes to be accessed in less than *n*/2 mutations.

The above approximations are confirmed by the computational results shown in figure 4, and are in line with the results of Ferrada and Wagner for RNA secondary structure and the HP model of protein folding, as well as with the Polyomino model [7].

### 2.4. Robustness versus frequency

It has been demonstrated that the phenotypic robustness in biological GP maps is much higher than one would expect for randomly distributed phenotypes [17–19], and scales roughly logarithmically with frequency [5,7]. The Fibonacci GP map exhibits the same relationship of robustness versus frequency (figure 5). The reason for this is straightforward: phenotypic robustness is *r*_p_ = 1 − (*l*/L) and the normalized phenotypic frequency is *f*_p_ = 2^−*l*^. We therefore have
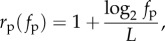
which describes the logarithmic relationship observed in figure 5.

## 3. Discussion and conclusion

Recently, one of the first empirical GP maps was studied by constructing the genotype networks for the binding site repertoires of 193 transcription factors in yeast and mice [20]. This work showed that transcription factors with large binding site repertoires have binding sites that are more robust and evolvable. As the degree to which mutations affect the binding affinity of a site strongly depends on the mutated position in the binding site, transcription factor binding sites contain constrained and unconstrained sequences. They therefore provide another example of a GP map that exhibits some of the properties discussed above for the Fibonacci, Polyomino and RNA GP maps, as well as genotypes with constrained and unconstrained parts. It would therefore be very interesting to investigate this empirical GP map more closely in the light of the sequence constraints and their effect on the GP map.

The Fibonacci GP map is arguably the simplest possible GP map with genotypes that contain coding and non-coding sequences. Nevertheless, it exhibits many of the properties that have been observed in the far more complex, and biologically realistic RNA secondary structure GP map, as well as in other evolutionary models, such as the Polyomino GP map. This implies that these structural properties of the maps are a result of the bimodal distribution of sequence constraints. In the Fibonacci GP map, the coding part is heavily constrained, while the non-coding part is completely free to mutate. As discussed above, the coding and non-coding parts of real genomes are constrained to a less definitive extent, but the bimodal nature of sequence constraints, both in RNA [5,11,12] and in form of the fundamental division of genomes into genes and intergenic sequences, and exons and introns, is undisputable. Importantly, the boundary between coding and non-coding sequences is itself part of the sequence—the ‘stop-codon’ in the Fibonacci GP map. In RNA, the arrangement with lowest free energy determines the bonds that form in the secondary structure, which means that in RNA this boundary is not a defined subsequence, but is nevertheless altered directly by mutations of the sequence that change a sequence position from a stem to a loop, or vice versa, because changes in the sequence alter the optimal thermodynamic arrangement of the molecules. The fact that, in all the GP maps discussed in this paper, the boundary between coding and non-coding sequences is subject to mutations is the likely reason for one of the most crucial properties of biological GP maps, namely the positive correlation between phenotypic evolvability and robustness [3], which explains how organisms can be both robust and evolvable. As the analytical calculation of this relationship in the Fibonacci GP map shows explicitly, the most important contribution to *e*_p_ comes from the terms that represent the possibility of the stop codon mutating. The Fibonacci GP map therefore offers strong evidence that the sequential nature of biological information determines the fundamental structure of GP maps, which in turn has a profound impact on the course of biological evolution. It also provides an analytical framework for the further study of the relationship between GP maps and evolution.

## Supplementary Material

Fibonacci GP map properties

## Supplementary Material

Polyomino (S_3,8) GP map shape space covering

## Supplementary Material

RNA GP map shape space covering

## Supplementary Material

Polyomino (S_3,8) GP map data for Figs. 2,3,5

## Supplementary Material

RNA GP map data for Figs. 2,3,5
